# Response to COVID-19 vaccination imaged by PD-L1 PET scanning

**DOI:** 10.1186/s41824-024-00196-7

**Published:** 2024-06-07

**Authors:** Michael P MacManus, Tim Akhurst, Sharon R Lewin, Fiona Hegi-Johnson

**Affiliations:** 1 Department of Radiation Oncology, Peter MacCallum Cancer Centre, 305 Grattan Street, Melbourne, Vic 3000 Australia; 2 The Sir Peter MacCallum Department of Oncology, The University of Melbourne, Melbourne, Australia; 3 Department of Molecular Imaging, Peter MacCallum Cancer Centre, Melbourne, Australia; 4 Department of Infectious Diseases, The University of Melbourne at the Peter Doherty Institute for Infection and Immunity, Melbourne, Australia; 5 Victorian Infectious Diseases Service, Royal Melbourne Hospital at the Peter Doherty Institute for Infection and Immunity, Melbourne, Australia; 6 Department of Infectious Diseases, Alfred Hospital and Monash University, Melbourne, Australia

**Keywords:** Positron emission tomography, Vaccination, COVID-19, PD-L1, Lung cancer

## Abstract

**Background:**

During a phase 0 clinical trial of an investigational programmed cell death ligand-1 (PD-L1) PET tracer in patients with non-small cell lung cancer (NSCLC), three patients received booster doses of COVID-19 vaccines before PD-L1 imaging.

**Methods:**

Five patients underwent whole-body PET/CT imaging with a novel PD-L1 tracer, constructed by attaching ^89^Zr to the anti PD-L1 antibody durvalumab. Intramuscular (deltoid) booster doses of mRNA BNT162b2 COVID-19 mRNA vaccine were coincidentally given to three patients in the month before PD-L1 tracer injection.

**Results:**

Two recently-vaccinated patients, in remission of NSCLC and receiving non-immunosuppressive cancer therapies (immunotherapy and tyrosine kinase inhibitor respectively), showed increasing PD-L1 tracer uptake in ipsilateral axillary lymph nodes. No asymmetric nodal uptake was seen in a third recently-vaccinated patient who was receiving immunosuppressive chemotherapy, or in two patients not recently-vaccinated.

**Conclusion:**

Immune response to mRNA BNT162b2 vaccination may involve regulation by PD-L1 positive immune cells in local draining lymph nodes in immunocompetent patients.

**Trial Registration:**

This trial was registered with the Australian New Zealand Clinical Trials Registry. Registration number ACTRN12621000171819. Date of Trial Registration 8/2/2021. Date of enrolment of 1st patient 11/4/2021. URL of trial registry record: https://www.australianclinicaltrials.gov.au/anzctr/trial/ACTRN12621000171819.

## Key points

**Question** Does mRNA vaccination for COVID-19 cause changes in the immune system that involve the programmed cell death ligand-1 (PD-L1)?

**Pertinent Findings** Two of two immunocompetent lung cancer patients in remission had markedly increasing uptake in draining axillary lymph nodes on PD-L1 positron emission tomography (PET) scans after recent COVID-19 vaccinations. A third, immunocompromised patient did not exhibit increasing nodal uptake.

**Implications for patient care** Response to mRNA vaccination in immunocompetent patients appears to involve regulation by PD-L1 expressing immune cells. This knowledge could help in the design of better vaccines.

## Introduction

There is increasing interest in studying immune responses to cancer and investigating the mechanisms of responsiveness and resistance to immunotherapy by imaging the biodistribution of biomolecules labelled with positron-emitting radioisotopes. These tracers may also provide information on other immune-related processes, including responses to infection and vaccination. Metabolic imaging with ^18^F-fluorodeoxyglucose (FDG)-PET is widely used to image non-small cell lung cancer (NSCLC). In recent years, specific PET tracers have also been developed to image components of the immune system, to explore sensitivity and resistance to immune checkpoint inhibitors (ICIs) in cancer patients (Hegi-Johnson et al. 2022a, b). The programmed cell death ligand-1 (PD-L1) is commonly-expressed on cancer cells and on immune cells, including macrophages, dendritic cells and T lymphocytes. PD-L1 is a target for cancer immunotherapy and patients treated with ICIs for NSCLC have shown dramatic improvements in survival (Socinski et al. 2018; Antonia et al. 2018). The programmed cell death protein1 (PD1)/PD-L1 axis is also involved in the immune response to viral infections (Schonrich and Raftery 2019; Wykes and Lewin 2018) but its role in vaccination is unknown.

We report results of imaging of the vaccination response for patients who received a novel PET tracer targeted at PD-L1 (Wichmann et al. 2023) during a cancer imaging trial (Hegi-Johnson et al. 2022a, b), conducted during the COVID-19 pandemic. Coincidentally, three of five patients underwent PD-L1 PET imaging after the mRNA BNT162b2 COVID-19 vaccine (Comirnaty, Pfizer/ BioNTech) was administered by deltoid intramuscular injection.

## Materials and methods

The phase 0 ImmunoPET trial investigated safety and biodistribution of a novel PET tracer intended for imaging tumor PD-L1 in cancer patients. The ethics committee-approved trial was registered with the Australian and New Zealand Clinical Trials Registry (Number 12,621,000,171,819) and all patients gave written informed consent. The tracer comprises a monoclonal antibody to PD-L1, used in cancer treatment, (durvalumab, Astra Zeneca (AZ), Wilmington, DE) bound to the positron-emitting isotope ^89^Zr (half-life 78 h) (Wichmann et al. 2023; Rudd et al. 2016). An intravenous injection of 70 megabecquerels (MBq) of ^89^Zr-labelled durvalumab is followed by serial PET/CT imaging in the following 5–6 days, allowing time for target binding and elimination of unbound tracer. Eligible patients had advanced (stage IV) PD-L1 positive IV NSCLC but were not required to have active tumor at the time of imaging.

## Results

Five patients were recruited (Hegi-Johnson et al. 2023), of whom three coincidentally received COVID-19 mRNA vaccines in the month before tracer administration. PD-L1 imaging results in relation to vaccination are described below by trial identifier number and summarized in Table 1. Table 2 shows changes in maximum standardized uptake value (SUVmax) in right and left axillary nodes at each time point for all patients.

**Table 1:** PD-L1 PET imaging in relation to vaccine booster dose administration

Patient Number	COVID-19 Booster Type	COVID-19 Booster date	PD-L1 PET tracer date	Vaccination - PD-L1 PET tracer interval	Axillary Node uptake?
1	NA	Not Vaccinated	Sep 9th 2021	NA	No
2	BNT162b2	Sep 26th 2021	Oct 19th 2021	23 days*	No
3	BNT162b2	Feb 2nd 2022	Mar 1st 2022	27 days	Yes
4	ChAdOx1n	July 8th 2021	Mar 16th 2022	8 months	No
5	BNT162b2	June 5th 2022	June 8th 2022	3 days	Yes

*Had received carboplatin 500 mg and pemetrexed 500 mg on October 4th 2021, before PD-L1 tracer injection on October 19th

**Table 2:** Sequential SUVmax in right and left axillary nodes over time

Patient Number	Day 1	Day 2	Day 3–4	5–7	Asymmetric Node uptake visualised ?
1 Right Left	2.2 1.83	0.81 2.2	2.2 1.7	2.3 1.8	No
2 Right Left	0.61 1.0	1.2 3.1	1.0 2.3	1.8 3.3	No
3 Right Left	3.7 4.5	4.2 5.8	4.7 7.2	6.0 **12.9**	Yes
4 Right Left	2.1 3.6	2.2 2.4	1.7 2.8	2.6 2.6	No
5 Right Left	3.2 0.8	2.3 3.2	2.7 4.0	2.9 **5.5**	Yes

Both patients with asymmetric axillary nodal uptake showed steadily increasing uptake in left axillary nodes over time, highlighted in Bold, on the same side as recent COVID-19 vaccination. Other patients did not show this pattern

### Patient 3

**Fig. 1:**
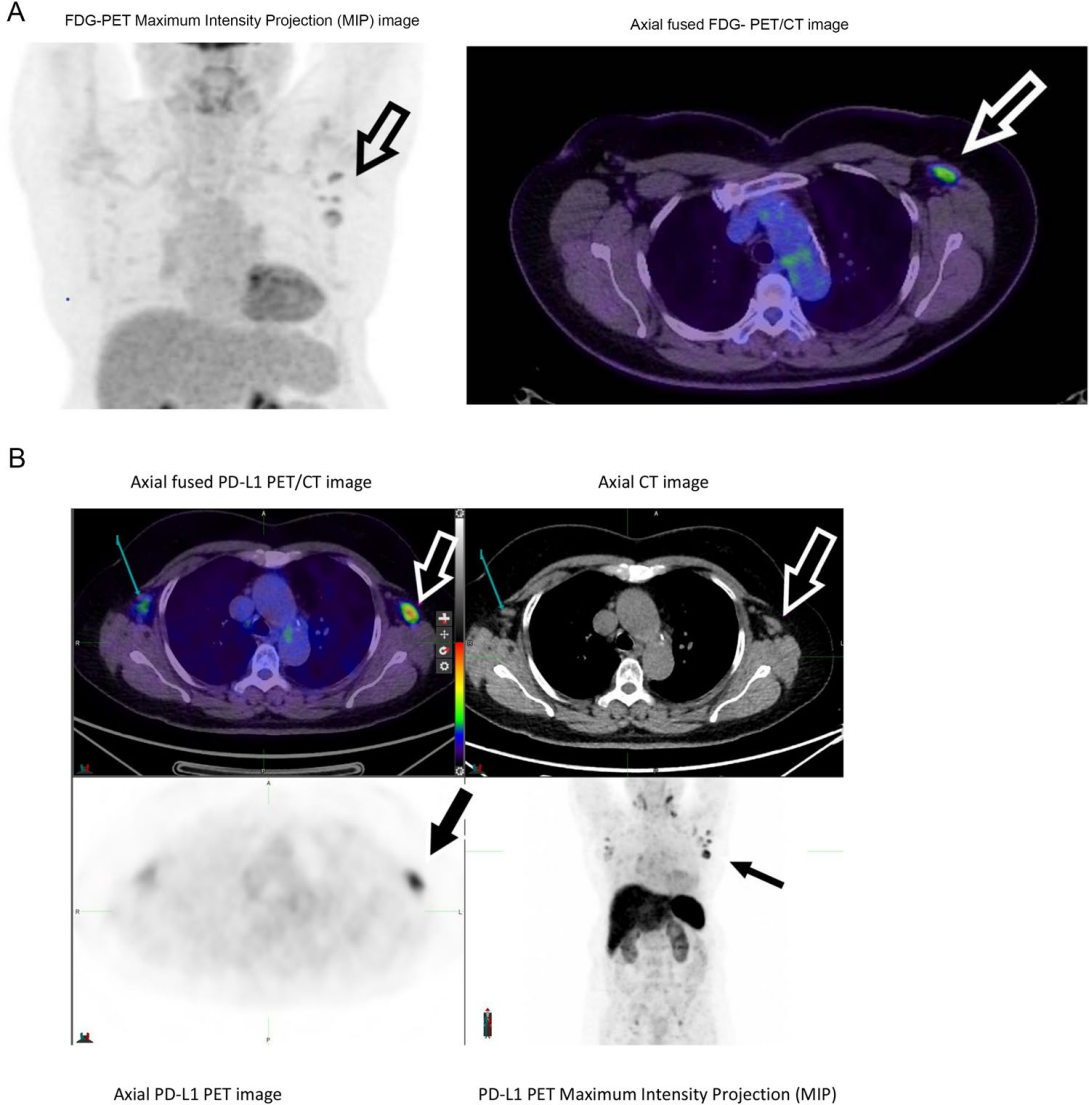
Patient 3 after COVID-19 vaccination, left deltoid muscle. (**A**) FDG-PET scan. Left axillary lymph nodes are indicated by arrows. (**B**) PD-L1 PET scan day 5. Left axillary lymph nodes are indicated by large arrows

A female aged 58, diagnosed with stage IV lung adenocarcinoma, had nodal and pulmonary metastases on baseline FDG-PET staging in January 2021. An EGFR mutation conferred sensitivity to tyrosine kinase inhibitor (TKI) therapy and she commenced osimertinib (AZ) on January 18th 2021. FDG-PET on August 31st 2021 showed a complete response. Mildly FDG-avid reactive left axillary nodes were reactive to ChAdOx1 nCoV-19 vaccination (Vaxzevria, CSL, Australia), in the left deltoid muscle on July 7th 2021. A second ChAdOx1 nCoV-19 vaccination occurred on September 14th 2021. A third, left deltoid vaccination, BNT162b2 (comirnaty, Pfizer/BioNTech) occurred on February 2nd 2022. Follow-up FDG-PET scan on February 25th 2022 showed remission of NSCLC and FDG-avid left axillary lymphadenopathy, consistent with an inflammatory response to vaccination (Fig. 1a).

She enrolled in the ImmunoPET study and received ^89^Zr-durvalumab on March 1st 2022, 27 days post vaccination. Serial PET/CT scans recorded the evolving biodistribution of the PD-L1 tracer. No accumulation of the PD-L1 tracer was observed previous tumor sites, consistent with ongoing remission. However, steadily increasing tracer uptake, characterised visually (Fig. 1b), and by sequential SUVmax (Table 2), peaking on the day five scan (Fig. 1b), occurred in the left axillary lymph nodes. Increasing PD-L1 tracer uptake was also observed in the contralateral axilla and bilateral groins, although less intense than in the left axilla (day 5 SUVmax 12.9 left axilla, 6.0 right axilla).

### Patient 5

**Fig. 2:**
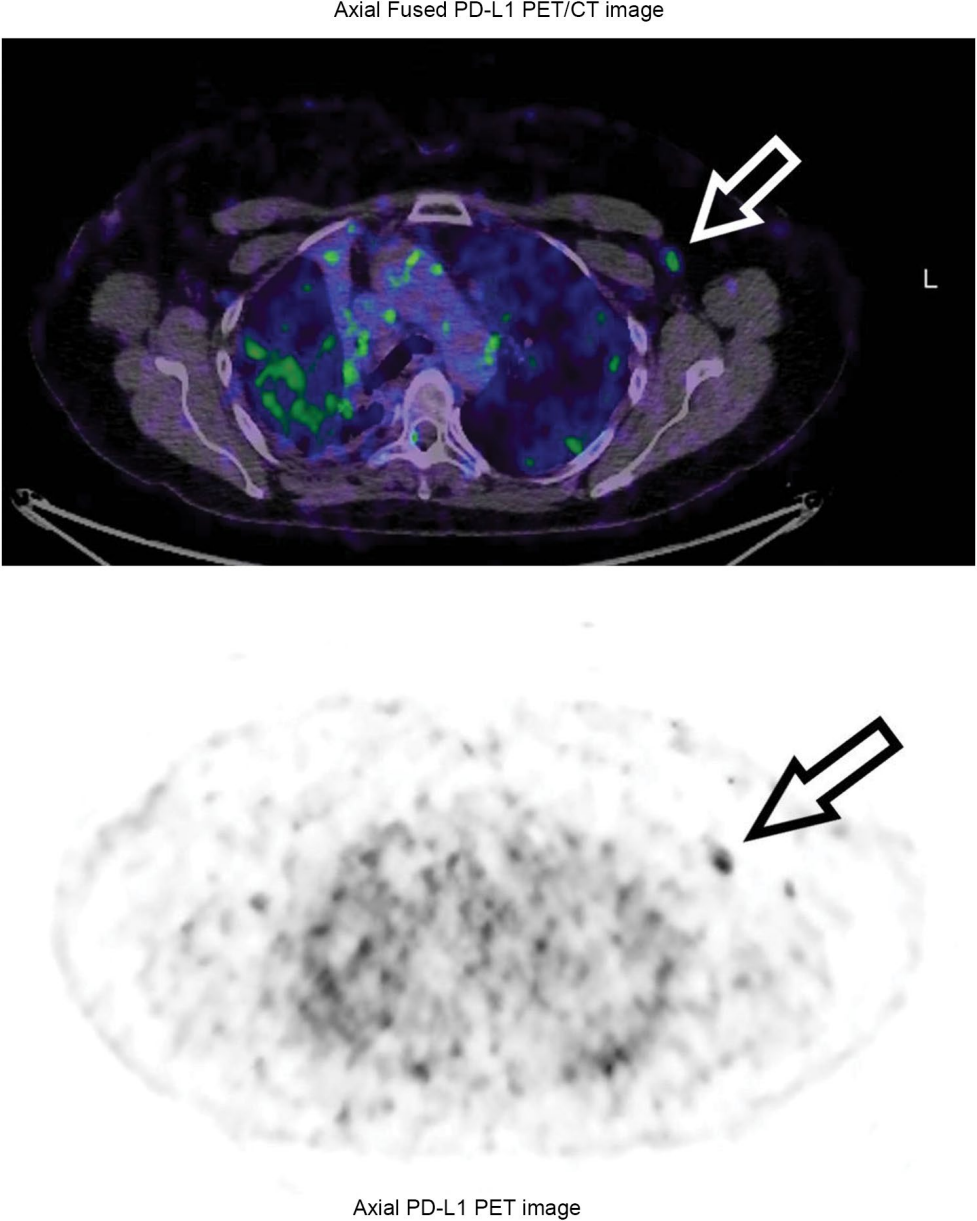
Patient 5 after COVID-19 vaccination left deltoid muscle. PD-L1 PET scan, day 4. Left axillary lymph node uptake is indicated by arrows

A female ex-smoker, age 63, had a large lung tumor with extensive nodal involvement was diagnosed with Stage IV adenocarcinoma of the lung in November 2019. Following radiotherapy in November 2019, she commenced ICI therapy with pembrolizumab (Merck, Rahway, NJ) in December 2019. She entered a sustained complete remission, documented by serial FDG-PET scans. She received two doses of ChAdOx1n CoV-19 vaccine in 2021. On June 5th 2022 she received a booster with the BNT162b2 COVID-19 vaccine into the left deltoid. On June 6th 2022 an FDG-PET scan showed no evidence of lung cancer but mild FDG-uptake at the deltoid vaccine injection site. She entered the ImmunoPET trial and received the PD-L1 tracer on June 8th 2022, 3 days after vaccination. On serial PD-L1 PET scans, increasing tracer uptake was observed in the left axillary nodes, peaking on day 5, characterised visually (Fig. 2), and by sequential SUVmax (Table 2). No other pathological uptake was observed.

The remaining trial patients showed neither significant asymmetrical uptake of PD-L1 tracer in axillary nodes nor steadily increasing SUVmax, as follows;

### Patient 2

A 46 years old male, diagnosed with stage IV adenocarcinoma of the lung in July 2020, received chemotherapy with carboplatin and pemetrexed, then maintenance pemetrexed and pembrolizumab. On October 18th 2021 he received adrenal radiotherapy for oligo-progressive disease and was enrolled in the ImmunoPET study. He received a second deltoid dose of Comirnaty vaccine on September 26 2021. Carboplatin 500 mg and pemetrexed 500 mg were given on October 4th 2021. PD-L1 tracer injection was on October 19th 2021, 22 days after vaccination. PD-L1 PET scans showed uptake in the adrenal tumor, without significant axillary uptake despite recent vaccination.

### Patients 1 and 4

Neither had received a COVID-19 vaccination within 3 months of the PD-L1 PET scan. Significant focal axillary nodal uptake was not observed.

## Discussion

We observed ipsilateral axillary PD-L1 tracer uptake after vaccination in two patients who were in remission of their lung cancers and were receiving non-immunosuppressive therapies, (osimertinib Ramalingam et al. 2020) and the ICI pembrolizumab respectively (Pardoll 2012). Steadily-increasing SUVmax was measured in nodes draining the site of recent deltoid injections of COVID-19 mRNA vaccines. A third, recently-vaccinated, patient in this study, who showed no significant asymmetric axillary uptake of PD-L1 tracer, had active cancer and received immunosuppressive chemotherapy around the time of imaging. Neither asymmetric axillary nodal uptake nor steadily-increasing axillary nodal SUXmax were observed in two patients who underwent PD-L1 imaging without recent vaccination.

PET-tracer uptake in draining lymph nodes after vaccination has not previously been reported with PD-L1 PET imaging. However, nodal uptake has been reported with FDG-PET (glucose), ^68^Ga-DOTATATE PET (somatostatin receptor), ^68^Ga-prostate-specific membrane antigen (PSMA, folic acid metabolism) (Orevi et al. 2022) and ^11^C-Choline (choline transporter-like protein–1) PET imaging (Schroeder et al. 2021) after COVID-19 vaccinations. Vaccine-related nodal PD-L1 activity detected on PET is potentially much more informative than non-specific nodal uptake due to glucose uptake or folic acid metabolism. PD-L1 is expressed on specific immune-related mononuclear cells, including dendritic cells, B-cells and NK cells (Wu et al. 2019). Germinal centre B cells are important for the generation of high-quality spike protein-specific antibodies following COVID-19 mRNA vaccination, and vaccination has been associated with increased intranodal and circulating T follicular helper cells that express high levels of PD-1 (Locci et al. 2013; Lederer et al. 2022). A compensatory upregulation of PD-L1 in lymphoid tissue to limit this immune response may be expected but has not been previously measured in people. Without correlative biopsies, it would be impossible to know with certainty whether the axillary uptake that we observed after vaccination is a specific finding related to immunity. However, the increasing SUVmax over time in both of our cases is more consistent with specific binding of tracer to PD-L1 positive cells in axillary nodes than with non-specific change due to blood flow or other physiological factors.

The differing vaccine-related findings in our cohort suggest that PD-L1 plays a role in the vaccine response. Both recently-vaccinated patients with cancers in remission and without a history of immunosuppressive therapy had asymmetric and increasing axillary uptake of the PD-L1 tracer suggesting that their imaging results might represent a normal vaccination response. In contrast, the lack of asymmetric axillary uptake in the recently-vaccinated patient with active metastatic cancer who was receiving cytotoxic chemotherapy may represent a subnormal immune response. PD-L1 PET imaging is in its infancy and physiological variations due to processes other than cancer have not been comprehensively described. When unexplained asymmetric and increasing axillary nodal uptake is observed on PD-L1 PET scans when no tumour is present, consideration should be given to vaccination as a potential cause.

New generations of PET tracers are being developed to explore the immune microenvironment of cancer. However, immune PET imaging could be repurposed to illuminate key cellular components of the total body immune response following vaccination. The ability of whole-body PET imaging to identify the anatomic location of processes involved in the immune response and to portray the evolution of the immune microenvironment over time could increase our understanding of the biology of vaccination and produce better vaccines.

## Data Availability

Relevant datasets used and/or analysed during the current study are available from the corresponding author on reasonable request. Will be made available on request
